# Single-Pixel Photon-Counting Imaging Based on Dual-Comb Interferometry

**DOI:** 10.3390/nano11061379

**Published:** 2021-05-24

**Authors:** Huiqin Hu, Xinyi Ren, Zhaoyang Wen, Xingtong Li, Yan Liang, Ming Yan, E Wu

**Affiliations:** 1 State Key Laboratory of Precision Spectroscopy, East China Normal University, Shanghai 200241, China; 52152000011@stu.ecnu.edu.cn (H.H.); 52190920021@stu.ecnu.edu.cn (X.R.); 18817216919@163.com (Z.W.); xtli625@163.com (X.L.); 2 Shanghai Key Laboratory of Modern Optical System, University of Shanghai for Science and Technology, Shanghai 200093, China; yanliangSPD@163.com; 3 Collaborative Innovation Center of Extreme Optics, Shanxi University, Taiyuan 030006, China

**Keywords:** photon counting, dual-comb imaging, spectrum-encoded, single-pixel, Fourier transform spectroscopy

## Abstract

We propose and experimentally demonstrate single-pixel photon counting imaging based on dual-comb interferometry at 1550 nm. Different from traditional dual-comb imaging, this approach enables imaging at the photon-counting regime by using single-photon detectors combined with a time-correlated single-photon counter to record the returning photons. The illumination power is as low as 14 pW, corresponding to 2.2 × 10^−3^ photons/pulse. The lateral resolution is about 50 μm. This technique paves the way for applying dual-comb in remote sensing and imaging.

## 1. Introduction

The development of single-photon detectors has revolutionized photon-counting laser ranging and imaging in speed and sensitivity [1,2,3,4,5,6,7,8,9,10]. Recently, there has also been interest in new methods that leverage the dual-comb target-encoded spectrum to perform imaging using a single-pixel detector [11,12,13,14,15,16]. In a general dual-comb spectroscopy system, a fast photodetector is usually used to receive the dual-comb signal. Since each comb is a mode-locked laser with numerous precisely equally spaced longitudinal modes, which are the so-called “comb lines”, a comb of radio-frequency beat notes will be observed at the output of the photodetector due to the interference of the dual-comb. Thus, the Fourier transform of this radio-frequency signal will reveal the spectral properties of the comb at optical frequency regime. This dual-comb spectroscopy based on Fourier transform has been widely used not only in tackling molecular dynamics but also in monitoring air quality in the atmospheric sciences [17,18]. Especially, by encoding the spatial information of the target to the broadband spectrum of the dual-comb, a three-dimensional profile of the sample object could be acquired with high precision. This so-called dual-comb imaging method, combining two optical combs of slightly different line spacing, converts an optical spectrum into a radio frequency (RF) electrical signal, encoding the optical signal comb with target spatial information into these radio frequency components. To retrieve the image, the spectrum that contains the spectrally encoded spatial information can be measured as a temporal waveform by the single-pixel photodetector. To map the different optical frequencies into distinct spatial locations, spatial dispersers, such as virtually imaged phase arrays (VIPA) [11,14] and/or gratings [13,16], are used. For example, this high-speed, multiplex imaging method has been demonstrated for confocal laser microscopy in life science [11,15] and has shown great potential for nanomaterial structure imaging [19]. Up to now, this method has only been applied using powers of the order of milliwatts to microwatts, which does not reach a photon-counting level [11,13,15,16]. However, to extend this method to more application scenarios such as remote imaging or sensitive biological tissue imaging, a dilemma will be encountered when the light signal reaches a photon-counting level [5,10]. To solve this problem, we come up with the idea to integrate the dual-comb imaging with a time-correlated single-photon counting technique which may provide the possibility to achieve both high resolution and high detection sensitivity in a dual-comb target-encoded imaging system for weak-light applications.

In this paper, we employed the dual-comb Fourier transform spectroscopy to retrieve the single-photon level signal, which carries spectrally encoded spatial information around 1550 nm. The experiment was conducted at an extremely low photon flux level, and we utilized a single-pixel single-photon detector together with a time-correlated single-photon counter (TCSPC) to detect the temporal interferogram waveform of the target-encoded reflected photons. A two-dimensional image was obtained by scanning the target with the dual optical frequency comb at a photon flux of 2.2 × 10^−3^ photons/pulse and lateral resolution of about 50 μm. Compared to a conventional dual-comb imaging system that uses a normal fast photodetector with the sensitivity at about milliwatts to microwatts [11,13,15,16], this dual-comb Fourier transform spectroscopy imaging system sensitivity could be as high as a few-photon level. Thanks to the high efficiency and low noise of the single photon-counting devices employed in the imaging system, the laser power required could be much reduced, leading to lower stray light noise in the system. We believe this work set the basis for the application of dual-comb single-photon interferometry, not only in remote spectrum-encoded imaging but also in biological tomography.

## 2. Experiment and Results

**Figure 1:**
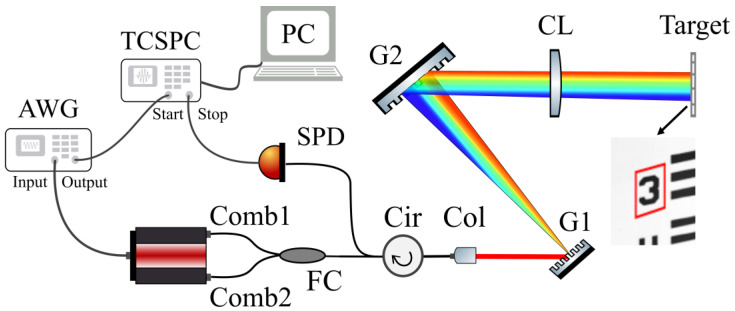
Experimental setup for the photon-counting dual-comb interferogram imaging. FC: 50:50 fiber coupler; Cir: fiber circulator; Attn: fiber attenuator; Col: collimator; G1, G2: a pair of reflective diffraction gratings with 600 lines/mm; CL: cylinder lens; SPD: single-photon detector; AWG: arbitrary waveform generator; TCSPC: time-correlated single-photon counter; Target: Positive 1951 USAF target marked with a red box as the laser illuminated area; PC: computer.

Dual-comb spectroscopy due to its unique spectral properties such as broadband spectra, uniformly spaced frequency distribution, long-term coherence, high resolution, and stability, has been demonstrated to be a powerful tool for many different areas. The time-correlated single-photon counting technique, combined with a single-photon detector, can improve the dual-comb imaging system photon sensitivity and can reduce counting noise, and enhance the image quality. Based on this idea, we designed the photon-counting dual-comb interferogram imaging experiment. The experimental setup is sketched in Figure 1. The light source consisted of two sets of electro-optic (EO) comb generators (WTEC-01-25, Optical Comb Inc., Tokyo, Japan) driven at slightly different frequencies of *f*_rep1_ (=25 GHz) and *f*_rep2_ (= *f*_rep1_ + Δ*f*_rep_, where Δ*f*_rep_ = 500 kHz), respectively. The comb carrier-envelope frequency was centered at *f*_eo_ = 192.8 THz, and one of the combs was shifted by Δ*f*_eo_ = 40 MHz using an acousto-optic modulator for avoiding frequency aliasing in dual-comb measurements [20]. The two combs had an identical spectrum ranging from 1543.02 to 1567.21 nm (or 191.29 to 194.29 THz), and each embraced *N* = 120 comb lines. Thus, the frequency of the *n*th comb line of a comb could be written as *f*_eo_ ± *n*·*f*_rep_ (or *f*_eo_ + Δ*f*_eo_ ± *n*·*f*_rep_), where *n* ≤ *N*/2 (*n* is an integer). Then, the two combs were combined by a 50:50 fiber coupler and collimated to the free-space by a fiber collimator after passing through a variable fiber attenuator and a fiber circulator. For spectral encoding imaging, the dual-comb beam was spatially dispersed with a pair of gratings (600 lines/mm) and then focused on a target in vertical direction by a cylindric lens with a focal length of 100 mm. The target was a commercialized resolution test chart with positive patterns (R3L3S1N—Positive 1951 USAF Test Target, Thorlabs) fabricated by vacuum depositing chrome patterns on the glass substrate as a reflective film. The target was placed on the focal plane of the cylinder lens. Consequently, a long strip-shaped area on the target was illuminated. The spatially dispersed light established a one-to-one correspondence between the spatial locations and the spectral elements (i.e., the comb lines) in a one-dimensional spectrum. Therefore, mechanically scanning the target in the direction vertical to the dispersed incident plane led to a two-dimensional (2D) image of a portion of the target (e.g., the area surrounded with the red box in Figure 1). The dual-comb beam was properly adjusted so that the reflection from the target would return along the same route to the collimator. By means of the fiber circulator, the reflected photons were guided to a single-photon detector for dual-comb heterodyne detection. For recording the dual-comb signal, a TCSPC system (Qutang, STDC-100, Qutools, München, Germany) was employed. The start channel of the TCSPC was triggered by the output of an arbitrary waveform generator (AWG, DG4162, Rigol, Suzhou, China) synchronized by the electrical timing reference from the dual-comb source. For correlated photon counting measurements, the output from the single-photon detector was fed to the TCSPC stop channel. In order to resolve spectrally the comb lines, the TCSPC time window should be longer than 1/Δ*f*_rep_ (i.e., recording more than one dual-comb interferogram). In our case, the AWG output (also the start signal) was set to Δ*f*_rep_/8 (=62.5 kHz), and correspondingly, the time window was eight times the dual-comb period.

**Figure 2:**
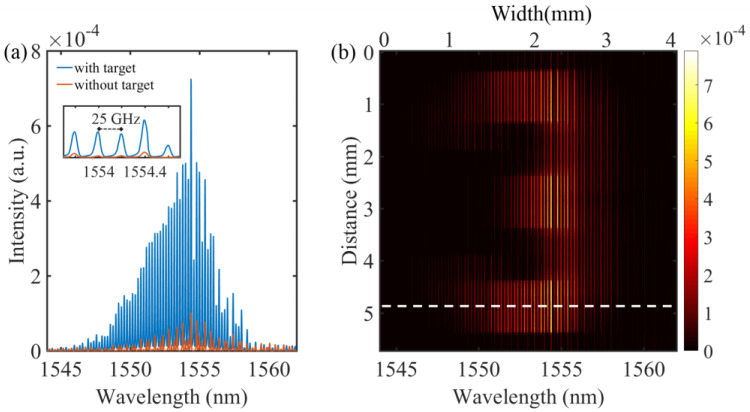
(**a**) A line spectrum obtained by the optical spectrum analyzer with and without the photon signal reflected from the target. Inset: zoom-in on the comb modes. (**b**) Two-dimensional spectrum encoding image of the target obtained by an optical spectrum analyzer. The blue spectrum in (**a**) was acquired at the position marked by the white dashed line in (**b**).

We first used an intensedual-comb beam (with weak attenuation) for testing and measured spectrum-encoded images with an optical spectrum analyzer (AQ6375B, Yokogawa, Tokyo, Japan). The wavelength resolution of the spectrometer was set to 0.05 nm, and the sampling resolution was set to 0.01 nm. The laser output power at the fiber collimator was about 1.745 mW and the returning laser power acquired by the spectrometer varied from 6 to 46 μW when scanning the different locations on the target. We scanned the target vertically with a step size of 0.05 mm close to the vertical beam size at the focus spot. For example, Figure 2a showed the spectra acquired at two different scanning situations. When we scanned the coated area of the target, the comb light was efficiently reflected, leading to an intense spectrum, as shown by the blue curve in Figure 2a. Otherwise, a strongly attenuated spectrum (the red curve) was recorded when we scanned on a transparent area of the target. Note that the current spectral resolution was sufficient for directly resolving the large longitudinal modes of the combs (spacing at ≈25 GHz). Therefore, the individual comb lines were displayed in Figure 2a. The comb modes could be seen clearly in the inset of Figure 2a. The spacing between two comb modes around 1552 nm is about 0.2 nm, corresponding to 25 GHz, which is exactly the mode spacing of the comb.

With the spectrum-encoding technique, we could get 2D spatial information of the targeted area. An example is shown in Figure 2b. To construct the image, we used a background spectrum recorded for a non-patterned area (i.e., the blue curve in Figure 2a) subtracting the scanned spectra. The color bar represents the relative reflectivity of the illuminated area from lowest (black) to highest (white) values. The bright strips in Figure 2b were caused by the comb structure of our light source. The lateral resolution (≈50 μm) here was limited by the comb line spacing. Optical combs with much finer line spacing could be used for spatial resolution enhancement. In our proof-of-concept experiment, two EO combs were chosen because of their excellent mutual coherence and long-term stability, benefiting dual-comb TCSPC measurements that necessitated coherent photon accumulation [2,21].

**Figure 3:**
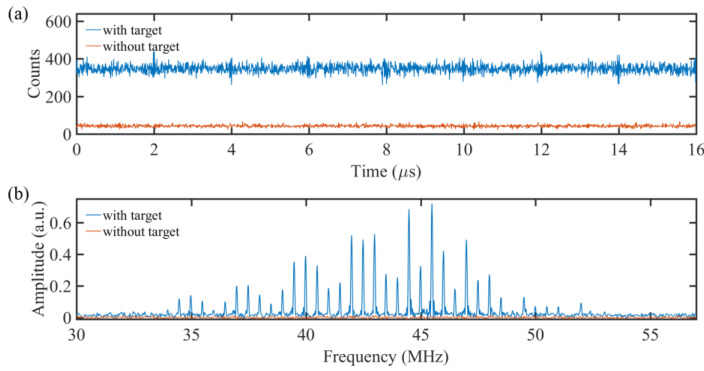
(**a**) Experimental photon-counting dual-comb interferogram detected by the SNSPD with and without the target. The raw data without filtering. The time bin width of the TCSPC was set to 1 ns, and the integration time was 1 s. (**b**) Fourier transform spectra of the photon-counting interferogram of the dual-comb interference with and without the target.

Then, the output laser power at the fiber collimator was attenuated to a few photons/pulse level. The light power after the collimator was 14 pW, corresponding to 2.2 × 10^−3^ photons/pulse. At this point, the returning photon flux was too weak for the optical spectrum analyzer to be detected. We replaced the optical spectrum analyzer with a single-photon detector for the 2D dual-comb imaging at photon-counting level. Firstly, we used a superconducting nanowire single-photon detector (SNSPD, LCDW16069, Photon Technology Co., Ltd., Shanghai, China) in the system as the photon receiver. The photon detection probability of the SNSPD was about 78% around 1550 nm, and the dark count noise was about 32 counts/s. The counting rate of the detector varied from 8.3 × 10^4^ counts/s to 8 × 10^5^ counts/s when scanning the light across the target. The output signal from the single-photon detector was sent to the stop channel of the TCSPC. Figure 3a shows the correlation between the returning photons and the triggering signal recorded by the TCSPC. This correlation curve reflects the photon-counting dual-comb interferograms. The sampling coincidence window was set to 1 ns, the exposure time was set to 100 ms, and the accumulation time was 1 s. Since the repetition rate of the trigger signal for the AWG was set to 62.5 kHz, corresponding to Δ*f_rep_*/8, we could observe 8 periods within 16 μs in the photon-counting dual-comb interferograms as shown in Figure 3a. To extract the spectral information, the time-domain dual-comb signals were zero-padded up to four-fold the number of digital samples and then Fourier transformed with triangular apodization [21]. The achieved spectral resolution could be calculated as 1.78/*t*_FFT_ *f*_rep1_/Δ*f*_rep_, where *t*_FFT_ was the apodization window of the interferometric data and 1.78 was a factor for triangular apodization [17,22]. In the experiment, the more periods we could acquire in a photon-counting dual-comb interferogram, the higher resolution we could obtain in the Fourier-transformed spectrum. Examples of two dual-comb spectra (corresponding to the recordings in Figure 3a) are displayed on a linear scale in Figure 3b. The signal-to-noise ratio (SNR) for a resolved comb line was technically limited by the photon counts and SNSPD dynamic range. For the measurement time of 1 s, the SNR for the highest comb line (the data in blue) at 45.5 MHz in Figure 3b was only 20:1, estimated by the line maximum divided by the standard deviation of a noise floor apart from the comb spectrum. Nevertheless, we recorded more than 30 comb lines (SNR > 1:1) for wavelength (or frequency) spatial mapping. Different from the usual spectrum-encoding image, here, a high-speed single-pixel detector without spectral resolution was employed, which may pave the way for a fast, compact portable spectrum encoding system. In addition, thanks to the single-photon detector, the sensitivity of our system reached a few-photon level (10^−18^ W). Consequently, the system will be a favorable platform for remote detection where the returning photon flux is quite low, such as in astronomical observation [23].

**Figure 4:**
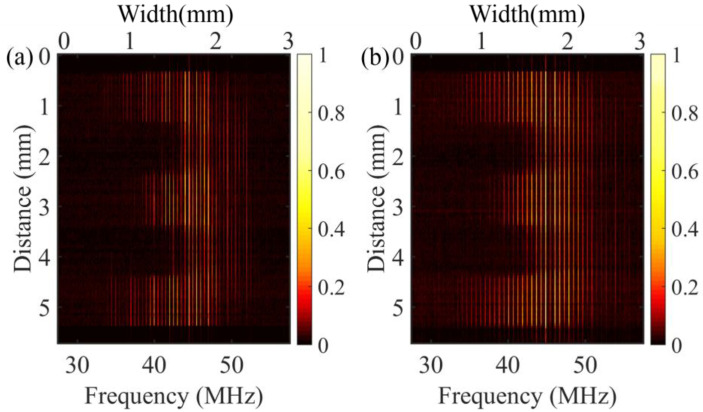
Two-dimensional photon-counting dual-comb interferogram of the target detected with an SNSPD (**a**) and an InGaAs-APD single-photon detector (**b**).

We implemented the dual-comb imaging at photon-counting level by scanning the target vertically in a stepwise manner. A 2D dual-comb spectrum-encoding image with spatial resolution of ≈50 μm is shown in Figure 4a. Compared with the image obtained by the optical spectrum analyzer, the image acquired by the dual-comb spectroscopy and SNSPD in the photon counting level shows the same scanning area with a similar pattern, indicating the reliability of the photon-counting dual-comb interferometric imaging system. We noticed that the image contrast of Figure 4a was lower compared to that of Figure 2b because of the limited SNR as well as the dynamic range of the single-photon detection and counting system. In addition, due to the same reason, the edges of the image, encoded with the frequency lines at the spectral edges of the combs, were lost. Using two optical combs with a flat-top spectrum or fewer comb line variations may improve the situation [20].

Furthermore, we compared dual-comb photon-counting imaging with single-photon detectors operating in different modes. We replaced the SNSPD with an InGaAs-APD (PGA-300-1, Princeton Lightwave, Cranbury, NJ, USA). Different from the SNSPD, which was operated in continuous mode, the InGaAs-APD was operated in gated Geiger-mode to reduce the dark count noise and the afterpulsing noise of the detector itself. The photon detection probability of the InGaAs-APD was about 7%. The repetition rate of the gating pulse was 1.25 GHz, and the dark count noise was about 1.2 × 10^3^ counts/s. The 2D dual-comb image acquired by the InGaAs-APD detector is shown in Figure 4b. We have to mention that the photon detection probability of the InGaAs-APD was approximately 11 times lower than the SNSPD. Even so, the recorded image had almost the same quality as the one with SNSPD thanks to the suppression of detector noise. The nearly identical images show the reliability of the photon-counting dual comb imaging system. Although the photon detection probability, dark count noise, and even the operation mode of the single-photon detector is quite different, the time-correlated photon counting could record correctly the temporal interferogram waveform, which lays the foundation of this photon-counting dual comb imaging. Moreover, other kinds of single-photon detectors could also be applied to this system to extend the application of this system to more different wavelengths, such as single-photon frequency upconversion detectors for the mid-infrared wavelengths.

## 3. Conclusions

In conclusion, by combining a single-pixel single-photon detector together with a time-correlated single-photon counter (TCSPC) in a dual-comb target-encoded imaging system, we achieved two-dimensional imaging at the single-photon level with both high precision and high detection sensitivity at 1550 nm. The 2D image was recovered by line scanning the target with the dual-comb laser light at the few photon level, showing great potential in the remote imaging where the transmission loss could not be ignored. Unlike in general spectrum encoding, there is no dispersing element in the detection part in the dual-comb Fourier transform spectroscopy imaging system, enhancing the mechanic stability of the imaging system. This method may have the potential to find widespread applications in nanomaterial imaging where the incident light should be very weak to avoid the damage of the sample.

## Data Availability

The data used to support the findings of this study are available from the corresponding author upon request.
